# The Certificate of the Certifying Surgeon

**Published:** 1921-03-12

**Authors:** 


### LEGAL QUIBBLES IN WORKMEN'S COMPENSATION.

### The Certificate of the Certifying Surgeon.

The judgment of the House of Lords in Corsar *v.* Archibald Russell, Ltd. (December 17, 1920), the full official report of which is now available, deals with a point not only entirely novel, but of great interest to practitioners.

A workman has the right to recover compensation in respect of certain diseases contracted in the course of his employment. One of these is "ulceration of the corneal surface of the eye, due to tar, pitch, bitumen, mineral oil, or paraffin, or any compound product or residue of any of these substances." A condition of the right to compensation is that the workman should obtain a certificate from the certifying surgeon for the district that the workman is suffering from the disease (Workmen's Compensation Act, 1906, Section 8, Sub-section (1)). There is an appeal to the medical referee, but, subject to the result of such an appeal, the certificate is conclusive.

### A Case in Point.

The workman in the present case was in the employment of the appellants as a labourer at their colliery, and worked at breaking up blocks of pitch for the manufacture of briquettes. He left his employment on April 21, 1919, because of the condition of his eyes, arising from his work. He went to the Glasgow Eye Infirmary, and his right eye was removed on June 9, 1919, in consequence of the disease. On July 3, 1919, he obtained from the certifying surgeon for the district the following certificate: "I . . . hereby certify that, having personally examined James Corsar on July 3, 1919, I am satisfied that he is suffering from a disease to which the Workmen's Compensation Act applies—namely, the disease mentioned in the schedule below, against which I have placed my initials, and is hereby disabled from earning full wages at the work at which he has been employed, and I certify that the disablement commenced on the twenty-first day of April, 1919." The disease in the schedule against which the certifying surgeon's initials are placed is "Ulceration of the corneal surface of the eye, due to tar, pitch, bitumen, mineral oil, or paraffin, or any compound product or residue of any of these substances." As "leading symptoms of disease," the surgeon certified: "He has lost his right eye as the result of corneal ulceration." The employers appealed against this certificate to the Medical Referee, and he, on July 15, 1919, sent the following letter to the Sheriff-Clerk, who communicated it to the parties: "James Corsar appeared before me yesterday. I regret, however, that I am not in a position to give a decision regarding the certificate of disablement given him by Dr. J. H. Murray on July 3, 1919. The certificate bears that Corsar is suffering from ulceration of the corneal surface of the eye, but as his right eye (the one affected) has been removed by operation, I cannot, of course, say whether the enucleation was performed for ulceration of the cornea." The appeal to the Medical Referee was dismissed by him on August 11.

The workman applied for compensation under the Act to the Sheriff-Substitute at Stirling, who, as arbiter, found that the certificate of the certifying surgeon was not a certificate of disablement as required by the Act, on the ground that as the eye had been removed it was impossible that the workman should, at the date of the certificate, be suffering from ulceration of that eye, and that as the eye had ceased to exist there could have been no personal examination of it. That decision was reversed in the Court of Session, and the employer now asked on this appeal that the judgment of the Court of Session should be set aside and that of the Sheriff-Substitute—as arbiter—restored. The questions thus were whether the certificate was bad on the ground that as the eye had been removed it was impossible that the workman could, at the date of the certificate, be suffering from ulceration of its corneal surface, and whether the certificate was vitiated by the fact that on the face of it it could not have been given upon personal examination of the eye, as that had been removed.

### Objections Fail.

The Supreme Court has now held that both these objections fail. The workman had lost his right eye in consequence of the disease. It was argued that at the date of the certificate he was not suffering from an industrial disease, but from the loss of the eye which had been removed in consequence of the disease. This objection could be sustained only upon far too narrow a reading of the Act and rule. The words "suffering from an industrial disease," in their fair reading, must be held to include a case in which the workman is suffering from the result of a surgical operation which the disease rendered necessary. The second objection was also considered unfounded. Though the form of the certificate under the rules shows that a personal examination of the workman is necessary, there is nothing to show that there must have been an examination of the diseased organ. Such an examination is impossible if the diseased organ had been removed in consequence of the disease before the examination. The certifying surgeon is quite entitled to take into account any other evidence which a medical man would take into account in making up his mind as to what had been the nature of the disease in the eye. He is entitled to hear the statements of the man himself, and to inquire of the surgeon who had removed the organ the facts as to the conditions which rendered its removal necessary. In all probability the certifying surgeon did make such inquiries before he gave the certificate.

### The Broad Interpretation.

It will be seen that the objections put forward were of an extremely technical nature, and one is glad that the House of Lords has so emphatically decided in favour of a broad interpretation of the statute—as Lord Dunedin tersely observed—"The function of the certificate is to assert that the man has had in the statutory sense an accident because of a disease which in a popular sense is not an accident. The actual form which has been issued by the Department is, I think, somewhat unhappily phrased, but I do not think it would be right by a slavish adherence to its literal form to defeat its proper purpose under the Act"; and more forcibly still Lord Shaw remarked: "The attack made upon the certificate is so meticulous as this: that because the eye has had to be removed in consequence of the disease mentioned, namely, ulceration of the cornea, yet, nevertheless, the patient cannot with propriety be said to be suffering from the disease, although he is suffering and must suffer all his life from the loss of his eye."

